# Malignancy-Associated Microangiopathic Hemolytic Anemia Mimicking Thrombotic Thrombocytopenic Purpura in a Patient With Metastatic Breast Cancer: A Case Report

**DOI:** 10.7759/cureus.115520

**Published:** 2026-08-31

**Authors:** Otiko Tcheishvili, Avtandil Mgeladze, Ana Eradze, Nino Kantaria

**Affiliations:** 1 Internal Medicine, New Hospitals, Tbilisi, GEO; 2 Intensive Care, New Hospitals, Tbilisi, GEO

**Keywords:** adamts13, hemolytic uremic syndrome (hus), metastatic breast cancer, microangiopathic hemolytic anemia, oncologic emergency, plasmic score, schistocytes, thrombotic microangiopathy, thrombotic thrombocytopenic purpura

## Abstract

Microangiopathic hemolytic anemia (MAHA) is a form of Coombs-negative hemolysis characterized by mechanical fragmentation of erythrocytes, producing schistocytes on the peripheral blood smear. When accompanied by thrombocytopenia and neurological features, the presentation closely resembles thrombotic thrombocytopenic purpura (TTP), a hematological emergency requiring urgent plasma exchange. However, advanced malignancy can produce an almost identical thrombotic microangiopathy (TMA) that responds poorly to plasma exchange, and for which the definitive treatment is control of the underlying cancer.

We report a 77-year-old woman with metastatic breast cancer and extensive osseous metastatic disease who presented with nausea, palpitations, generalized weakness, and transient neurological symptoms. Investigations revealed severe anemia, moderate but rapidly progressive thrombocytopenia, markedly elevated lactate dehydrogenase, elevated indirect bilirubin, undetectable haptoglobin, a negative direct antiglobulin test, and schistocytes on the blood smear. Brain magnetic resonance imaging showed acute bilateral frontal ischemic infarctions. Although early coagulopathy and active cancer yielded an intermediate PLASMIC score, TTP could not be excluded. Empiric plasma exchange and high-dose corticosteroids produced no hematological improvement, and the patient died shortly thereafter. ADAMTS13 testing was delayed by a pre-analytical error. The clinical course and lack of response favored malignancy-associated MAHA with disseminated intravascular coagulation over TTP.

This case underscores that in patients with disseminated malignancy, MAHA with thrombocytopenia and neurological signs should raise early suspicion of cancer-associated TMA, even while TTP is treated empirically.

## Introduction

Thrombotic microangiopathy (TMA) is a clinical syndrome with diverse etiologies, defined by microangiopathic hemolytic anemia (MAHA), thrombocytopenia, and organ injury from microvascular thrombosis. The unifying morphological hallmark is the schistocyte, a fragmented erythrocyte generated as red cells are sheared within partially occluded microvessels [1,2].

The most feared cause is thrombotic thrombocytopenic purpura (TTP), which results from a severe deficiency of ADAMTS13, the metalloprotease that cleaves von Willebrand factor multimers [3]. Untreated TTP is frequently fatal, and because confirmatory ADAMTS13 testing is often unavailable at the point of care, guidelines support immediate empiric plasma exchange (PLEX) whenever TTP is clinically suspected [4,5].

Malignancy can produce a clinically and laboratory-wise similar picture, as both conditions share a final common pathway of microvascular occlusion leading to mechanical erythrocyte shearing and platelet consumption. Both TTP and cancer-associated TMA may cause MAHA, thrombocytopenia, and ischemic organ injury, including neurological manifestations, making them difficult to distinguish clinically. Cancer-associated TMA arises through mechanisms including widespread microvascular tumor emboli, bone marrow infiltration (carcinomatosis), and disseminated intravascular coagulation (DIC), and has been described in association with various advanced solid tumors, such as mucin-producing adenocarcinomas of the breast and stomach [6-8]. Unlike TTP, it typically shows little or no response to PLEX, and prognosis depends on control of the underlying malignancy [6,7,9].

Clinical prediction models such as the PLASMIC score aid in rapidly stratifying the likelihood of severe ADAMTS13 deficiency [10]. However, distinguishing TTP from malignancy-associated TMA remains particularly difficult in resource-limited settings, where ADAMTS13 activity testing requires international shipment and results can be delayed by days [11]. We describe a patient with metastatic breast cancer in whom MAHA, thrombocytopenia, and acute cerebral infarction mimicked TTP, and in whom the absence of response to PLEX pointed to malignancy-associated TMA as the underlying process [6,8,9].

## Case presentation

A 77-year-old woman presented to the hospital with nausea, palpitations, and generalized weakness that had begun the previous evening and progressively worsened over several hours. She additionally reported two transient neurological episodes: the first, the previous evening, involved numbness of the tongue and right limb with dysarthria that resolved spontaneously within approximately five minutes; a second similar episode occurred on the day of admission and also resolved spontaneously. There was no loss of consciousness, chest pain, dyspnea, or persistent focal neurological deficit at presentation. Her history was notable for metastatic breast cancer. The primary tumor - a hormone receptor-positive, HER2-negative (Luminal B) invasive ductal carcinoma - had originally been diagnosed more than two decades earlier and treated with mastectomy and adjuvant chemotherapy. A locoregional recurrence in the supraclavicular lymph nodes had been treated with chemotherapy, and distant metastases to the liver and bone had subsequently been identified. Her most recent imaging demonstrated extensive progressive osseous metastatic disease involving the sternum, ribs, scapula, pelvis, and vertebrae. No bone marrow biopsy was performed during this admission. She had received multiple lines of endocrine and targeted therapy, including tamoxifen, fulvestrant, the cyclin-dependent kinase 4/6 inhibitor ribociclib, and bisphosphonate therapy.

She had been discharged one day before the current presentation following inpatient management of anemia and thrombocytopenia thought to be related to chemotherapy-associated bone marrow suppression, at which time her hemoglobin was 8.5 g/dL and platelet count was 47 × 10⁹/L, with no active complaints. On examination, she had multiple petechiae over the trunk and limbs. Initial laboratory studies demonstrated severe anemia (hemoglobin 4.8 g/dL; mean corpuscular volume (MCV) 88 fL) and thrombocytopenia (87 × 10⁹/L). Peripheral blood smear demonstrated schistocytes. Hemolysis was supported by elevated total and indirect bilirubin, reticulocytosis (4.5%), and undetectable haptoglobin (<10 mg/dL), with a negative direct antiglobulin test. Lactate dehydrogenase (LDH) measured on Day 4 was markedly elevated at 1,412 U/L. Coagulation studies demonstrated a progressive coagulopathy, with the international normalized ratio (INR) increasing from 1.49 on admission to 2.18 on Day 4 and 2.15 on Day 6, while fibrinogen declined from 2.12 g/L on Day 4 to 0.87 g/L on Day 6. These coagulation abnormalities raised the possibility of concurrent DIC.

Subsequent brain MRI, performed to evaluate the transient neurological symptoms, demonstrated acute bilateral frontal ischemic infarctions. While these findings are clinically concerning for microvascular ischemia in this context, a definitive microangiopathic etiology cannot be established by imaging alone, and other embolic or thrombotic mechanisms cannot be excluded. Creatinine initially measured 120 µmol/L, decreased to 92 µmol/L on Day 4, and subsequently increased to 167 µmol/L by Day 6. The evolution of key laboratory values is summarized in Table 1.

**Table 1:** Serial laboratory findings during hospitalization *The platelet count of 31 × 10⁹/L on Day 6 was measured following platelet transfusion. Abbreviations: aPTT, activated partial thromboplastin time; INR, international normalized ratio; LDH, lactate dehydrogenase; MCV, mean corpuscular volume; PT, prothrombin time

Parameter	Day 1	Day 4	Day 5	Day 6	Reference range
Hemoglobin (g/dL)	4.8	6.9	6.8	6.0	12.0-15.5 g/dL
MCV (fL)	88	89	-	-	80-100 fL
Platelets (×10⁹/L)	87	16	9	31*	150-400 ×10⁹/L
INR	1.49	2.18	1.45	2.15	0.8-1.2
PT (s)	16.4	24.1	15.9	23.7	11-14 s
aPTT (s)	35.4	32.5	23.8	35.1	25-35 s
Fibrinogen (g/L)	-	2.12	1.31	0.87	2.0-4.0 g/L
Creatinine (µmol/L)	120	92	-	167	45-84 µmol/L
Total bilirubin (µmol/L)	74	90	107	-	<21 µmol/L
LDH (U/L)	-	1,412	-	-	135-225 U/L

A peripheral blood smear (fingerstick sample) revealed schistocytes - including helmet cells and triangular and crescent-shaped erythrocyte fragments - at an estimated 5-7 per high-power field (~4-6% of erythrocytes on manual review), suggestive of microangiopathic (mechanical) hemolysis (Figure 1).

**Figure 1:**
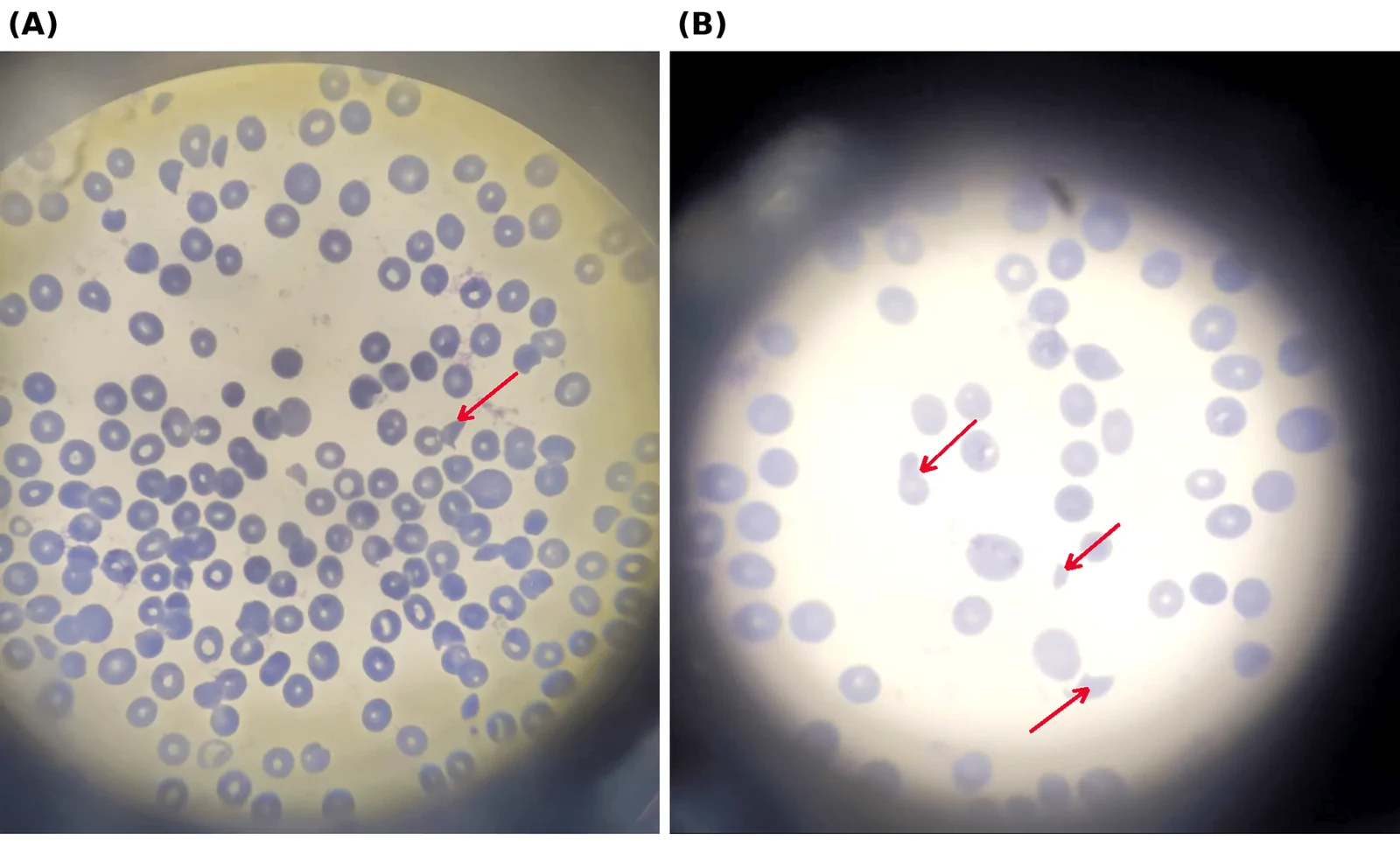
Peripheral blood smear demonstrating schistocytes Peripheral blood smear (fingerstick sample, Wright-Giemsa stain, 1000× oil immersion) from two separate fields showing schistocytes (red arrows), including helmet cells and triangular and crescent-shaped erythrocyte fragments, at an estimated 5-7 per high-power field (~4-6% of erythrocytes on manual review). This finding is consistent with microangiopathic (mechanical) hemolysis. (A) and (B) represent separate fields from the same smear.

Brain magnetic resonance imaging (MRI) with diffusion-weighted imaging demonstrated acute ischemic infarctions involving the right corona radiata and left basal frontal cortical region, together with periventricular leukoencephalopathy and cortical atrophy (Figure 2).

**Figure 2:**
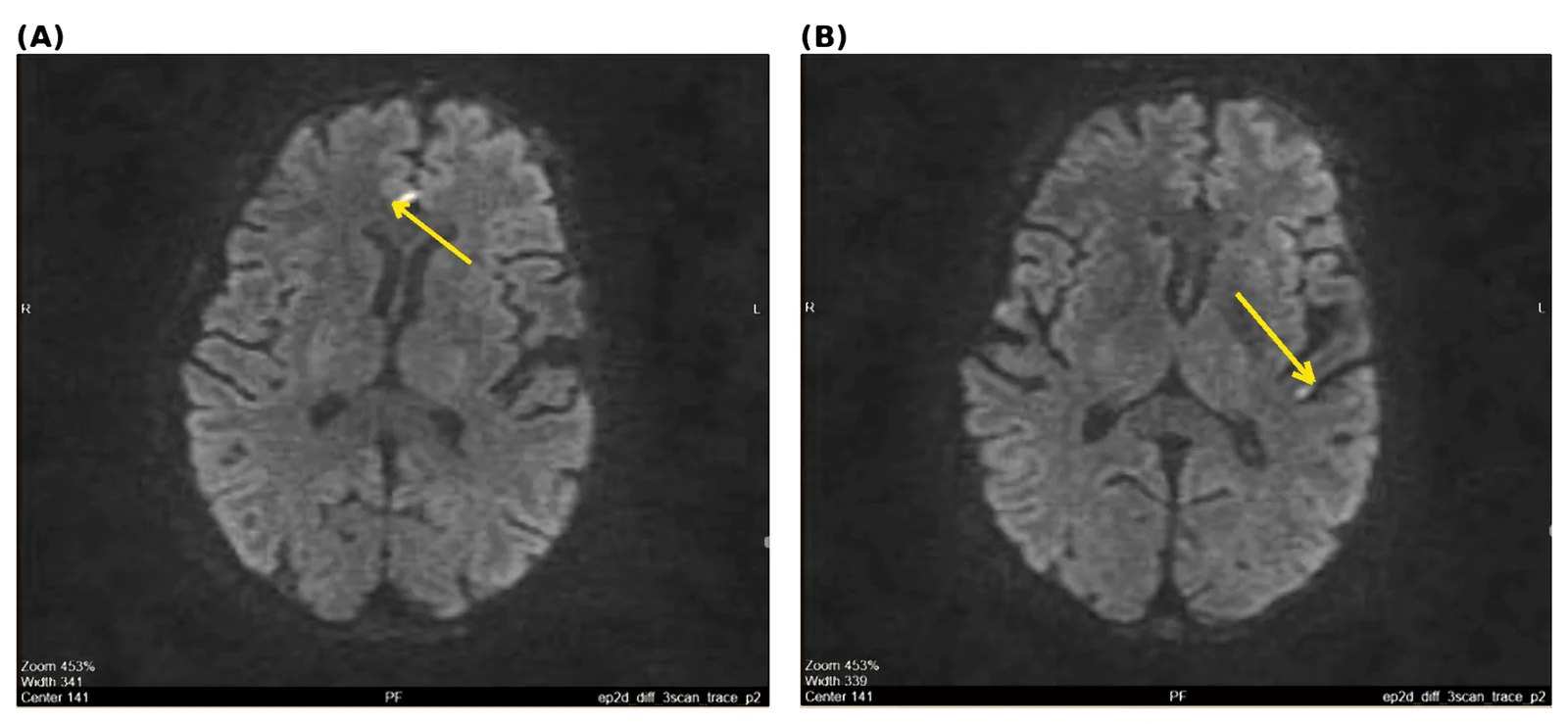
Diffusion-weighted MRI demonstrating acute bilateral frontal ischemic infarctions Brain MRI with diffusion-weighted imaging showing acute ischemic infarctions (arrows) involving (A) the right corona radiata and (B) the left basal frontal cortical region, with associated periventricular leukoencephalopathic changes and cortical atrophy. These lesions were temporally associated with the patient's transient neurological episodes in the setting of microangiopathic hemolytic anemia and severe thrombocytopenia; a mechanistic link to microvascular injury is plausible but cannot be established by imaging alone, and embolic or other thrombotic etiologies cannot be excluded.

The PLASMIC score was calculated on admission and was 5, corresponding to an intermediate probability of severe ADAMTS13 deficiency. Active malignancy and a platelet count >30 × 10⁹/L each contributed no points, while hemolysis, MCV <90 fL, INR <1.5, creatinine <2 mg/dL, and absence of prior transplantation contributed one point each [10]. Overall, her presentation appeared to represent a complex clinical overlap of potential pathological processes: chronic cytopenias from suspected chemotherapy-related marrow suppression and tumor infiltration, acute consumptive coagulopathy suspicious for DIC, and features of malignancy-associated TMA. However, because the combination of MAHA, progressive thrombocytopenia, and acute cerebral ischemia raised immediate concern for TTP - which is rapidly fatal if left untreated - empiric therapeutic PLEX and high-dose corticosteroids (intravenous methylprednisolone, escalated to 1000 mg daily) were initiated on Day 4, together with supportive transfusion of packed red cells.

Following standard management of suspected TTP, platelet transfusion was initially withheld in the absence of life-threatening bleeding [12]. A sample for ADAMTS13 activity was drawn prior to PLEX on Day 4 and sent to an international reference laboratory [5]. Despite the prompt initiation of PLEX and corticosteroids, there was no apparent hematological improvement: hemolysis persisted, and the platelet count declined further to a nadir of 9 × 10⁹/L on Day 5. The patient was admitted to the intensive care unit, where her course on Day 6 was complicated by life-threatening gastrointestinal hemorrhage from gastric mucosal erosion. At this juncture, because of active major bleeding and severe thrombocytopenia, platelet transfusion was administered alongside endoscopic adrenaline injection to achieve hemostasis [12,13]. Following transfusion, the platelet count transiently increased to 31 × 10⁹/L. Over the subsequent days, she developed worsening renal function, rising lactate, metabolic acidosis, and progressive cytopenias, ultimately dying during the hospitalization.

The initial ADAMTS13 sample drawn on Day 4 could not be analyzed because the citrated plasma had clotted, representing a pre-analytical limitation [11]. A subsequent ADAMTS13 sample was obtained before her death; however, the result, which became available several days after her death, indicated that the second specimen was also clotted and unsuitable for analysis. Neither specimen yielded a valid ADAMTS13 activity or inhibitor result. Consequently, TTP could neither be confirmed nor definitively excluded on the basis of ADAMTS13 testing.

## Discussion

This case illustrates the diagnostic challenge posed by MAHA in a patient with advanced malignancy. The presentation fulfilled the clinical criteria of a thrombotic microangiopathic process - Coombs-negative hemolytic anemia with schistocytes, thrombocytopenia, and end-organ neurological involvement - and closely mimicked TTP [1,2].

TTP is caused by a severe deficiency of ADAMTS13, leading to accumulation of ultra-large von Willebrand factor multimers, platelet aggregation, and microvascular thrombosis [3]. Because delayed treatment carries high mortality and confirmatory testing is frequently unavailable at presentation, the standard of care is to begin empiric PLEX as soon as TTP is suspected [4,5].

Malignancy-associated TMA is an important mimic. It occurs most commonly with disseminated mucin-secreting adenocarcinomas, and breast cancer is among the tumors most frequently implicated [6-8]. Proposed mechanisms include tumor microemboli obstructing the microcirculation, extensive bone marrow infiltration by carcinoma (carcinomatosis) with associated leukoerythroblastosis, tumor-related activation of coagulation, and overlapping DIC [6,7]. In this patient, radiologically documented metastatic breast cancer with documented progressive osseous disease, prior cytopenias, and evolving coagulopathy provided a strong clinical context for malignancy-associated MAHA [6,8]. Drug-induced TMA was also considered. However, there was no clear temporal relationship between the patient's anticancer therapies and the onset of MAHA and thrombocytopenia, while the progressive metastatic disease, suspected bone marrow involvement, and evolving coagulopathy provided a more likely clinical explanation for the overall picture.

Several features favored malignancy-associated MAHA/TMA with DIC overlap over immune-mediated TTP. First, early laboratory abnormalities, specifically an elevated INR and reduced fibrinogen level during the initial hospital course, were atypical of primary TTP and raised concern for a concurrent DIC/TMA process [6,7]. This was also reflected in the patient's intermediate PLASMIC score (5 points), which, while not low enough to strongly argue against TTP, fell below the high-risk range typically associated with severe ADAMTS13 deficiency; the score could not definitively exclude TTP, particularly in the setting of acute cerebral infarction [10]. To contrast these overlapping syndromes, the key clinical, laboratory, and therapeutic differences are outlined in Table 2. The absence of an apparent hematological response to adequate PLEX and high-dose corticosteroids further supported consideration of a non-immune TTP (iTTP) process, although lack of response to PLEX alone is not diagnostic of malignancy-associated TMA [6,7,9]. In contrast to iTTP, in which PLEX is generally associated with hematological improvement, cancer-associated TMA may remain refractory when the underlying malignant process is uncontrolled [6,7]. The leukoerythroblastic-type blood picture, rising white cell count, progressive metastatic disease, and evolving coagulopathy further supported malignancy-associated TMA/MAHA as the leading clinical consideration, although this diagnosis could not be definitively established [6,7].

**Table 2:** Clinical, laboratory, and therapeutic differences between immune TTP and malignancy-associated TMA Data collated and adapted from References [1,4,6,7,9,10]. Abbreviations: DIC, disseminated intravascular coagulation; INR, international normalized ratio; TMA, thrombotic microangiopathy; TTP, thrombotic thrombocytopenic purpura

Clinical or Laboratory Feature	Immune TTP (iTTP)	Malignancy-Associated TMA (Most Likely Clinical Diagnosis)
Primary pathophysiology	Autoantibody-mediated severe ADAMTS13 deficiency	Potentially multifactorial; tumor microemboli, possible bone marrow carcinomatosis, and overlapping DIC
ADAMTS13 activity	Profoundly reduced (<10%)	Typically normal or mildly reduced (non-severe deficiency); not interpretable in the present case
PLASMIC score	High risk (usually 6-7)	Intermediate risk (5); influenced by active malignancy and other clinical/laboratory parameters
Coagulation profile (INR/fibrinogen)	Usually normal; significant coagulopathy is uncommon at presentation	Abnormal; elevated INR and hypofibrinogenemia, consistent with DIC overlap
Response to plasma exchange (PLEX)	Generally associated with hematological improvement and platelet recovery	May be refractory when driven by active malignancy; no apparent hematological response was observed in this case
Definitive treatment	Therapeutic PLEX with immunosuppression (rituximab/corticosteroids)	Treatment directed at the underlying malignancy, together with supportive management of TMA/DIC

The case also highlights a systemic constraint. ADAMTS13 activity was not measured locally and required international shipment. The pre-analytical failure of both samples, with clotting of the citrated plasma, and the resulting inability to obtain an interpretable ADAMTS13 activity or inhibitor level reflect the real-world limitations of relying on esoteric assays during rapidly evolving hematological emergencies [11]. In resource-limited settings, clinicians may need to initiate PLEX for suspected TTP before definitive ADAMTS13 results are available. This reality reinforces the importance of integrating the clinical context, diagnostic scores, coagulation profile, and response to therapy while acknowledging the uncertainty that remains when confirmatory testing is unavailable [5,10,11].

Recognizing malignancy-associated TMA has direct management implications. While empiric PLEX remains appropriate when TTP cannot be safely excluded, definitive treatment of cancer-associated microangiopathy depends on controlling the underlying malignancy [6,8,9]. Continued PLEX in a patient with refractory, tumor-driven TMA - especially one complicated by severe DIC-related hypofibrinogenemia - may expose the patient to substantial procedural and bleeding hazards without treating the underlying pathological process [6,12]. Early recognition allows goals of care and the risks and benefits of further intervention to be discussed realistically with the patient and family [6,7].

This report has limitations inherent to a single case. ADAMTS13 activity was never interpretable during the patient's life, so immune-mediated TTP cannot be entirely excluded on laboratory grounds. The clinical features most strongly favored malignancy-associated MAHA/TMA with DIC overlap, based on the underlying metastatic malignancy, MAHA, progressive thrombocytopenia, coagulopathy/hypofibrinogenemia, and lack of an apparent hematological response to PLEX; however, these findings are not pathognomonic. Tissue confirmation of tumor microangiopathy, such as bone marrow biopsy demonstrating carcinomatosis, was not obtained during the brief and rapidly fatal course. Accordingly, malignancy-associated TMA should be regarded as the most likely clinical diagnosis rather than a definitively proven underlying process. Nonetheless, the case is instructive precisely because it reflects the real constraints under which such patients are managed.

## Conclusions

Malignancy-associated MAHA is a rare but life-threatening complication of advanced cancer that can closely mimic TTP. In a patient with metastatic breast cancer, the combination of Coombs-negative hemolytic anemia, severe thrombocytopenia, and acute neurological injury should prompt early consideration of cancer-associated TMA, particularly when bone marrow involvement and early coagulopathy are present. While empiric PLEX is justified whenever TTP cannot be excluded, an intermediate PLASMIC score together with a lack of hematological response should raise suspicion for a malignancy-driven process - though neither finding alone is diagnostic - for which definitive treatment is control of the underlying cancer. Careful attention to the clinical context and response to therapy remains essential to timely, realistic decision-making when confirmatory testing is delayed or unavailable, and malignancy-associated TMA should be regarded as the most probable rather than definitively established diagnosis in such cases.
